# Supplementary material for: Are carbon emissions trading and green financial instruments synergistic? -Comprehensive quantitative research based on content analysis
Source: PLoS One. 2024 Mar 7;19(3):e0298601. doi: 10.1371/journal.pone.0298601 (PMC10919601; doi:10.1371/journal.pone.0298601)
Supplement: S1 Data — (ZIP) [file pone.0298601.s001.zip › Data/地级市Z_F工作报告.zip!/┴╔─■/╔≥╤⌠╩╨/└┤╫╘╣1⁄2╓┌║┼í╛┬φ┐╦╩2╛▌═°í┐_2011.docx]

各位代表：

　　现在，我代表市政府向大会报告工作，请予审议，并请市政协各位委员提出意见和建议。

　　一、2010年及“十一五”时期主要工作回顾

　　过去一年，在省委、省政府和市委的正确领导下，市政府带领全市人民，深入贯彻落实科学发展观，扎实推进以“调结构、优环境、惠民生”为重点的各项工作，圆满完成了市十四届人大三次会议确定的各项任务。

　　预计全市地区生产总值实现5015亿元，比上年增长14.0%；全社会固定资产投资完成5007.4亿元，增长36.2%；社会消费品零售总额实现2062.5亿元，增长18.3%；地方财政一般预算收入完成465.4亿元，增长17.6%；城市居民人均可支配收入达到20540元，增长11.2%；农民人均纯收入达到10005元，增长14.3%。全市经济社会实现又好又快发展。

　　（一）经济综合实力进一步提升

　　工业结构调整步伐加快。规模以上工业增加值实现2361.4亿元，增长19.1%；实现利税660亿元，增长29.5%；规模以上装备制造业增加值占工业比重达到50.3%。机床、北方重工、远大等骨干企业不断发展壮大，沈鼓等国有企业改制重组取得新进展，宝马新工厂、米其林搬迁扩产等一批重大项目开工建设。编制完成七大战略性新兴产业发展专项规划，现代建筑产业园、泗水科技城等产业园区建设加快，我市成为国家节能与新能源汽车示范推广试点城市。

　　农业和农村经济快速增长。全面落实强农惠农政策，投入“三农”资金104.7亿元，增长13.7%。克服了洪涝等多重自然灾害影响，粮食总产量达到32.5亿公斤，其他主要农产品产量稳步增长。新增设施农业和高效特色农业面积44.4万亩，开工建设投资1000万元以上农事项目90个。县域产业集群不断发展壮大，县城和新城、新市镇建设步伐加快。县域特别是四县（市）经济增长速度明显高于全市平均水平。

　　服务业特别是现代服务业稳步发展。服务业增加值实现2242.2亿元，增长13.2%，现代服务业增加值占服务业比重达到52.8%。区域金融中心建设步伐加快，新增金融机构6家、小额贷款和融资担保机构25家。会展交易额实现1600亿元，旅游总收入实现528亿元，房地产业保持平稳健康发展。金廊核心段29个重点项目和东北总部基地等重大服务业项目加快推进。

　　自主创新能力和对外开放水平不断提高。全市规模以上高新技术产品增加值完成1006亿元，增长19.5%。并购国内外高科技企业10家，引进51个海外研发团队，东软集团软件架构新技术实验室和北方重工全断面掘进机实验室成为国家重点实验室，我市跻身国家创新型城市试点行列。4个世界500强企业和一批大型跨国公司投资项目签约落地，当年调资超千万美元项目84个，全市实际利用外商直接投资50.5亿美元。调入内资910亿元，增长68.1%。外贸出口实现42亿美元，增长20.0%。

　　（二）城市建设和管理得到加强

　　空间资源要素实现优化配置。全面修编城市总体规划，调整完善了区域发展规划和专项规划。实施行政区划调整和中心城区扩容，东陵区、浑南新区和航高基地“三区”实行合署办公。完善土地储备制度，严格实行净地出让，土地集中统一管理进一步加强。

　　“四大战役”取得明显成效。大力实施植树绿化工程，整修改造了一批道路桥梁。强化建筑市场监管，25家市政园林企业因施工质量、资质等问题被清出沈阳市场。开展“清洁沈阳”活动，城乡环境面貌进一步改善。加强道路交通综合治理，交通拥堵问题得到缓解。

　　浑南新城建设全面启动。编制完成新城总体规划和专项规划，启动道路、电力等基础设施建设，全面实施水系改造和绿化工程，农民回迁房建设加快推进，沈阳南站等重点项目开工建设，“十二运”比赛场馆及配套设施建设有序展开。

　　重大基础设施建设扎实推进。地铁1号线通车试运营，2号线建设进展顺利。启动南北二干线、二环、三环、四环等改造建设工程，沈阜、沈彰开发大道（一期）建成通车。沈阳站、沈阳北站改造和沈丹客运专线建设加快推进。大伙房水库输水配套工程（一期）竣工。沈、抚、铁三市共享024电信区号并网工程启动实施。

　　生态环境建设取得新进展。全国环境建设样板城首批27个示范工程项目加快推进，沈北新区、棋盘山开发区通过国家生态区验收。蒲河生态廊道建设取得阶段性成果，144公里景观路建成通车。实施重点企业节能技术改造和脱硫改造工程，建成5座污水处理厂，年度节能减排任务圆满完成。

　　（三）重点民生工程扎实推进

　　就业形势保持总体稳定。全市实名制就业20.7万人，以创业带动就业工作体系不断完善，就业困难家庭至少一人实现稳定就业，困难家庭大学生就业率达到100%，城镇登记失业率控制在3.2%。上调最低工资标准，稳步推进工资集体协商，劳动关系进一步和谐稳定。

　　社会保障体系日趋完善。出台了城镇职工基本养老保险关系转移接续办法。康平县新型农村社会养老保险改革试点稳步推进，有3.3万名农民领取了养老金。全市有10.8万被征地农民参加了养老保障。提高退休职工养老金标准、城乡低保标准和医疗保险待遇水平，新型农村合作医疗人均筹资标准达到165元。新建100个社区老年人日间照料站，市养老服务中心（一期）和市儿童福利院投入使用。市就业和社会保险综合服务平台正式运行。

　　城乡居民生活条件进一步改善。为4.67万户低保家庭和低收入家庭提供了住房保障，完成了389个弃管住宅区综合改造，实施了供暖热源建设和管网改造工程。加大扶贫开发力度，25个重点贫困乡（镇）农民人均纯收入增加810元，贫困人口减少3.7万人。新建农村饮水工程127处，解决了21.3万农民饮水安全问题。

　　（四）社会事业和民主法制建设取得积极进展

　　社会事业全面发展。完成200所接收外来务工人员子女学校改造，扎实推进城乡义务教育均衡发展，普通高中标准化建设不断推进，全民终身教育体系进一步完善，我市成为国家装备制造业职业教育试验区。医药卫生体制改革稳步推进，国家基本药物制度全面落实，市骨科医院、妇婴医院、儿童医院（一期）改扩建工程即将投入使用，市第一医院、精神卫生中心改扩建工程主体完工。沈阳艺术中心开工建设，大型现代评剧《我那呼兰河》荣获“文华奖”，艺术惠民“双百万”工程被评为国家文化创新工程。圆满完成“省运会”承办任务，并获得金牌、奖牌和总分三项第一。

　　社会管理水平不断提高。坚持用群众工作统揽新形势下信访工作，信访工作“沈阳模式”不断完善。加大社会稳定风险评估力度，最大限度防止侵害群众利益的行为发生。积极化解信访积案，坚决纠正侵害群众利益的行为。大力加强社区建设，社区综合服务功能进一步增强。深入开展食品药品和安全生产专项整治，严厉打击刑事犯罪，“平安沈阳”建设取得新进展。

　　民主法制建设进一步加强。依法行政深入推进，市政府提请市人大常委会审议了8件地方性法规草案，发布实施了8件政府规章。认真落实市人大及其常委会的决议、决定，自觉向市人大常委会报告工作、向市政协通报工作，主动接受市人大及其常委会的依法监督和市政协的民主监督。全年办理市人大代表建议848件、市政协提案570件，办复率达到100%。

　　国防动员教育、人防和民兵预备役工作进一步加强，双拥共建工作扎实推进。民族团结进步事业不断发展，对口支援工作成效显著。人口与计划生育、统计、监察、审计、参事、宗教、侨务、广播电视、新闻出版、税务、工商行政管理、气象、防灾减灾、档案、修志、文史、红十字会、慈善、残疾人事业和老龄工作等都取得了新成绩。

　　各位代表，2010年全市工作取得的成绩，标志着我们圆满完成了“十一五”规划确定的各项目标任务。回首“十一五”，沈阳老工业基地向全面振兴迈出了坚实步伐。

　　经济持续快速发展。地区生产总值、全社会固定资产投资和地方财政一般预算收入分别是2005年的2.1倍、3.7倍和2.6倍，地区生产总值和全社会固定资产投资双双突破五千亿元。

　　产业升级进程加快。装备制造等八大优势产业支撑作用进一步增强，增加值占工业比重达到80.3%。现代服务业加快发展，占服务业比重达到52.8%。现代农业发展迅速，设施农业和高效特色农业面积占耕地比重达到44.9%，畜牧业产值占农业比重达到50.3%。

　　发展空间不断优化。“东南西北中”五大发展空间格局基本形成，城市功能分区更趋合理，沈阳经济区一体化进程不断加快，发展空间进一步拓展。

　　城市面貌变化显著。城乡基础设施不断完善，综合服务功能明显提升。3个区（开发区）通过国家生态区验收，3个区（县）通过国家生态区（县）技术核查，生态市建设取得了积极进展。

　　改革开放迈出新步伐。沈阳经济区建设上升为国家战略，我市新型工业化综合配套改革全面启动。国有企业改革成效显著。非公有制经济加快发展，增加值占全市比重达到65.5%。新增世界500强投资企业30家，实际利用外商直接投资245.5亿美元。

　　和谐沈阳建设取得新成就。城乡居民收入分别是2005年的2.0倍和1.98倍，就业形势平稳，社会保障体系不断健全，社会事业全面发展。

　　各位代表，过去五年沈阳取得的可喜成绩，是省委、省政府和市委正确领导的结果，是全市人民齐心协力、拼搏奋斗的结果。在此，我代表市政府，向全市各族人民致以崇高的敬意！向人大代表、政协委员，向各民主党派、工商联、无党派人士和各人民团体，向驻沈人民解放军、武警部队官兵和中、省直单位，向所有关心、支持沈阳的港澳台同胞、海外侨胞和国际友人表示衷心感谢！

　　在肯定成绩的同时，我们也清醒地看到，当前我市经济社会发展中还存在一些问题。主要是经济运行质量和效益还不够高，具有牵动力的重大项目还不够多，城市建设和管理水平需要进一步提升，一些关系人民群众切身利益的问题亟待解决，经济发展环境有待进一步改善，政府自身建设还需进一步加强。对此，我们要在今后工作中着力加以解决。

　　二、“十二五”时期目标任务和2011年重点工作

　　各位代表，“十二五”时期是沈阳老工业基地实现全面振兴的关键期，也是难得的多重历史发展机遇的叠加期，我们必须进一步增强使命感，抢抓机遇，加快发展。根据《中共沈阳市委关于制定全市国民经济和社会发展第十二个五年规划的建议》，市政府组织编制了《沈阳市国民经济和社会发展第十二个五年规划纲要（草案）》，提请本次大会审议。

　　“十二五”时期，我市经济社会发展的指导思想是：以邓小平理论和“三个代表”重要思想为指导，深入贯彻落实科学发展观，解放思想，抢抓机遇，以科学发展为主题，以加快转变经济发展方式为主线，以改革开放为动力，深入推进做优发展空间、做大中心城市、做强县域经济、加强生态建设和着力改善民生“五大任务”，继续保持经济社会又好又快发展的强劲势头，推动沈阳老工业基地在科学发展道路上实现全面振兴，建成更高水平的小康社会。

　　总体目标是：建设国家中心城市、先进装备制造业基地、生态宜居之都，进而率先实现老工业基地的全面振兴。

　　主要预期目标是：全市地区生产总值年均增长12%以上，全社会固定资产投资年均增长10%以上，社会消费品零售总额年均增长13%以上，地方财政一般预算收入年均增长13%以上，城市居民人均可支配收入年均增长12%以上，农民人均纯收入年均增长13%以上，单位地区生产总值能耗和主要污染物排放总量按国家规定持续下降。

　　重点任务是：做优发展空间，切实将空间优势转化为产业优势和竞争优势；做大中心城市，不断增强中心城市的承载力、聚集力和辐射力；做强县域经济，加速城乡一体化发展；加强生态建设，建成国家生态市和全国环境建设样板城；着力改善民生，全面提高城乡人民生活水平。

　　各位代表，今年是“十二五”开局之年。我们要全面贯彻落实市委十一届十次全会精神，突出做好“抓项目促调整、抓环境促提升、抓民生促和谐”等重点工作，以增量带动结构优化，以创新促进产业升级，以发展保障民生改善，为实现“十二五”规划目标奠定基础。

　　今年全市经济社会发展主要预期目标是：地区生产总值增长12%，全社会固定资产投资增长15%，社会消费品零售总额增长15%，地方财政一般预算收入增长13%，城市居民人均可支配收入增长12%，农民人均纯收入增长13%，城镇登记失业率控制在4%以内，全市单位地区生产总值能耗下降2%，主要污染物排放总量均下降1.5%。

　　今年，我们要重点抓好以下八个方面工作。

　　（一）突出抓好重大项目建设

　　充分发挥项目建设对经济增长的拉动作用，以存量吸引增量，以增量促进结构优化和发展方式转变。

　　加大项目引进建设力度。着眼于积累发展后劲，下大气力引进投资规模大、增长潜力大、带动产业结构优化、支撑经济社会发展的重大项目。强化项目推进责任制，突出抓好一批新的重点项目开工，继续推进续建项目加快建设。

　　切实保障要素供给。努力提高土地集约节约利用水平，稳步推进城乡建设用地“增减挂钩”，做好土地储备和土地整理工作，确保重大项目用地需求。加强政企、银企对接，探索多种融资方式，吸引社会资本参与重点工程建设。

　　（二）着力提升工业发展水平

　　以“五项工程”为抓手，加快推进工业化与信息化融合，进一步提升工业整体水平和竞争力。全市规模以上工业增加值增长16%。

　　实施企业提升工程。加快西部装备制造、南部高新技术、东部汽车、北部通讯等四大产业聚集区发展，推进特种机床、机泵阀及模具等配套园区建设，积极培育知名品牌，支持重点骨干企业加快发展，建设具有工程总承包和系统集成能力的大型企业集团，争取新增4户产值超百亿企业。

　　实施项目工程。大力推进宝马新工厂、米其林搬迁扩产、机床产业园等重点项目建设，启动建设海尔工业园、沃得工程机械产业园等一批重大项目，推动在谈项目尽快落地。

　　实施并购工程。启动并购国内外项目40个、完成15个，力争在并购规模和掌控核心技术上取得突破，加快培育拥有关键核心技术、在国际国内市场具有较强竞争力的骨干企业。

　　实施产业集群工程。加快培育汽车及零部件、机床及功能部件、电气及配件、软件及电子信息、农产品精深加工、手机（光电）、通用及专用机械、医药化工、现代建筑和民用航空等10个千亿产业集群和22个百亿产业集群。今年，力争汽车及零部件产业集群产值突破1000亿，3个产业集群进入百亿产出行列。

　　实施节能与淘汰落后工程。启动50项重点节能工程，推广应用20项重点节能新技术、新产品，支持企业升级改造，加快淘汰落后产能，万元工业增加值能耗要降至1吨标准煤以下。

　　加快国家创新型城市建设。强化企业在技术创新中的主体地位，加强重点企业院士工作站建设，充分发挥驻沈高校和科研院所优势，促进产学研紧密结合，推动国家重大科技专项在关键技术和产业化上取得新突破。启动建设沈阳国家大学科技城。培养、引进和用好各类人才。支持新兴产业发展，加快现代建筑产业园、泗水科技城、航高基地、通航产业基地、康泰斯电子级多晶硅等重点园区和重点项目建设,积极推进节能与新能源汽车产业化。

　　（三）大力发展县域经济

　　认真落实强农惠农政策，努力做好“三农”工作，不断壮大县域经济，协调推进工业化、城镇化和农业现代化。

　　大力发展现代农业。加大农田水利设施建设力度，修建120座农用万亩桥，改造30万亩中低产田，努力提高粮食单产水平。抓好“菜篮子”工程，确保农产品有效供给。加快“一乡一业”、“一县一业”发展步伐，开工建设投资500万元以上农事项目120个，新增设施农业10万亩、高效特色农业20万亩、水产养殖小区40个、畜牧养殖小区100个。大力发展专业合作社和农民技术协会，加强农业技术推广和动植物疫病防控，推进农业标准化生产，支持农产品冷链物流发展，加强农产品出口基地建设，培育名牌产品和地理标志产品。加快光辉、九龙河现代农业示范园区发展，启动沈北新区国家种业研发基地建设。

　　加快推进县域工业化和城镇化。以产业园区为依托，以重大项目为牵动，加快产业集群建设，力争新民包印产业集群、辽中铸锻机加产业集群产出超百亿。突出抓好县城、新城、新市镇和重点中心镇规划建设，加强道路、电网等基础设施建设，推进农村环境综合整治，推广使用沼气等新型能源，改善农村生产生活条件。支持新民、辽中冲刺全国百强县，法库、康平在全省县域综合经济实力排名中位次前移。

　　进一步拓宽农民增收渠道。全面落实粮食直补等惠农政策，进一步优化种养结构，增加农民生产经营收入。大力发展农产品加工业和非农产业，加快农村剩余劳动力转移，增加农民工资性收入。加大农村扶贫开发力度，不断增加贫困地区农民收入。

　　（四）加快发展服务业

　　进一步壮大服务业规模，全面提高服务业特别是现代服务业发展水平，不断完善中心城市功能。服务业增加值增长12%。

　　着力推动产业集聚。推进重点服务业集聚区建设，力争北站金融集聚区、和平金融街、大中街商贸文化集聚区、太原街都市商贸中心、北一路钢材物流产业带等5个集聚区营业收入突破50亿元。进一步提升太原街、中街、东中街、长江街等商业街区集聚发展水平。

　　努力促进业态提升。大力发展现代服务业，注重发展生产性服务业。推进金融机构集聚和金融要素市场建设，加快建设东北区域金融中心。壮大重点物流园区和重点物流企业规模，加强商务会展设施建设和大型会展营销策划，铁西区全国服务业综合改革试点要取得积极进展。加快建设高力汽车博览城、雨润农副产品全球采购中心、五洲国际商贸城、永安国际物流港等一批服务业重大项目。金廊项目建设要全面提速。

　　积极实施品牌塑造。努力打造特色街区、专业市场和商贸品牌，进一步提升西塔、北市、中山路、沈阳路等民俗和特色商业街区发展水平，扩大五爱、南塔等专业市场的影响力，力争新增驰名商标5件、省市著名商标95件，扶持沈阳“老字号”品牌做优做大。

　　大力发展文化产业。不断壮大出版发行、娱乐演出、动漫制作等主导产业，积极培育创意设计、影视传媒、广告会展等新兴产业。注重发展生态旅游、乡村旅游、温泉旅游、工业旅游和特色文化旅游。加快浑南动漫产业基地、棋盘山国家文化产业示范区等重点园区建设。做大做强沈阳出版发行集团等大型文化骨干企业。支持辽宁广播电视传媒中心建设。启动建设工业博物馆，改造新乐遗址博物馆，加快沈阳艺术中心建设，华强文化科技产业基地（一期）投入运营。

　　（五）进一步加大城市建设和管理力度

　　以承办“十二运”为契机，加快新城建设和老城改造步伐，进一步改善城乡环境面貌。

　　加快“十二运”比赛场馆和配套设施建设。全面启动比赛（备用）场馆、训练场馆的新建和改扩建工程，加快运行中心、接待中心、新闻中心、媒体村、技术官员村和运动员村建设，确保“十二运”建设项目如期投入运行。

　　全力推进浑南新城建设。高标准建设主干路网、地下管廊以及水、电、气、热等基础设施和配套设施。支持桃仙机场T3航站楼、沈阳南站和省博物馆、科技馆、图书馆、档案馆等重点项目建设。开工建设同方联合商业综合体、宝龙商业综合体等一批重大项目。加快大浑南地区全域城市化步伐。

　　加快老城区改造提升。支持京沈、沈丹客运专线和沈阳新北站建设，加快南北二干线、二环快速路、三环高速路、四环快速路和一批跨浑河桥改造建设，推进地铁1、2号线延长线建设，争取2号线年底前通车，力争开工建设4号线、10号线。加快故宫方城等重点地区和零星棚户区改造，综合整治18个城市出入口地区，改造北站南广场和沈阳站广场，做好北站北出口广场建设前期工作。加快给排水设施和地下管网综合改造，实施雨污分流。进一步规划和开发利用好地下空间。

　　提高城市精细化管理水平。进一步调整完善城市建设管理的体制机制，强化区级主体责任，倡导“出门就上班”的工作作风，加强数字化城市管理。整修改造100条道路和17座桥梁，实施主要街路沿线绿地景观和重点公园改造提升。继续开展“清洁沈阳”活动，拆除违章建筑，规范户外广告和各种牌匾，进一步改善市容环境。大力发展智能交通，实施公交优先发展战略，调整增加公交线路和车辆，在主要街路和繁华商业街区建设一批过街天桥、地下通道和停车场。深入开展“迎全运文明沈阳行动”，提高城市文明程度和市民文明素质，为承办“十二运”创造良好的社会氛围。

　　扎实推进生态环境建设。大力发展循环经济和环保产业，加快建设全国环境建设样板城，力争苏家屯区、于洪区、辽中县通过国家生态区（县）验收。继续推进蒲河生态廊道建设，实施辽河、浑河、百里环城水系综合改造。大力推进城乡绿化和沈西北防沙治沙工程，全市植树造林58.3万亩。加快沈化、东药等高排放企业整体搬迁改造，加大企业脱硫改造力度，建设南部污水处理厂和西部污泥处置厂，扩建西部污水处理厂，积极推进中水回用。

　　（六）不断深化改革开放

　　充分发挥沈阳经济区建设上升为国家战略的政策优势，进一步深化改革、扩大开放，为经济社会发展注入新的动力。

　　加快综合配套改革。全面实施《沈阳市国家新型工业化综合配套改革试验方案》，深化沈北新区先导示范区、铁西区生产性服务业、金融商贸开发区优化金融生态、大浑南地区工业化与信息化融合、苏家屯区城乡统筹发展、辽中县现代综合保税物流创新等专项改革。携手其他兄弟城市共同促进沈阳经济区一体化发展，推进沈本、沈阜、沈彰开发大道（二期）和环经济区高速公路建设，加大沈阳经济区城际连接带19个新城、新市镇建设力度，加快李相新城、浑河新城、芝布特拉国际小镇和蒲河温泉小镇等重点项目建设。

　　深化重点领域改革。推动国有企业产权多元化、资本证券化，完成红梅集团重组，推进盛京银行等10户企业上市，争取我市成为首批“新三板”扩容试点城市。继续深化行政审批制度改革，推广“批管分离”。放大高新区体制机制优势，带动大浑南地区改革发展。积极推进公立医院改革试点，全面实现医药卫生体制改革三年目标。加强公共资源交易管理，进一步规范交易行为。

　　继续推进农村改革。规范农村土地承包经营权流转市场，逐步扩大林权抵押贷款范围和规模。继续深化供销合作社改革。支持农村投资担保公司发展，加快组建农村商业银行。

　　支持非公有制经济加快发展。鼓励民间资本进入金融服务、基础设施、社会事业等领域。积极帮助中小企业融资，支持中小企业技术改造，扶持本地企业发展壮大。

　　努力提高对外开放水平。进一步优化引资结构，更加注重引进高端装备制造业、高新技术产业、战略性新兴产业和现代服务业项目，以及世界500强、知名跨国公司、大型央企和行业领军企业项目，鼓励外资企业在沈设立地区总部和研发中心，实际利用外商直接投资增长5%，调入内资增长10%。支持优势产品、高新技术产品和自主品牌产品出口，推动加工贸易转型升级，出口总额增长10%。鼓励有实力的企业“走出去”，开展对外投资、工程承包和劳务合作。继续办好制博会等重大国际活动。

　　（七）全面加强社会建设

　　在实现经济又好又快发展的同时，着力保障和改善民生，大力发展社会事业，让全市人民共享经济社会发展成果。

　　切实做好就业和社会保障工作。加快国家创业型城市建设，鼓励、支持自主创业和自谋职业，加大就业培训和就业援助力度，帮助就业困难人员特别是零就业家庭人员实现就业，全市实名制就业20万人。继续推进集体合同“彩虹计划”，广泛开展工资集体协商，维护农民工合法权益，促进劳动关系更加和谐稳定。扩大社会保险覆盖面，提高社会保险待遇水平，实行城镇职工基本医疗保险门诊统筹，提高新型农村合作医疗人均筹资标准。建立和完善低保标准调整联动机制。加快建设区域性养老中心和社区老年人日间照料站。改造建设一批区域性残疾人托养、康复中心，争创“全国残疾人工作示范城市”。新建5000套公共租赁住房，进一步完善住房保障体系。继续实施弃管住宅区综合改造。加快推进城市供水二次加压系统改造和农村饮水工程建设，让城乡群众吃上放心水。

　　推进社会事业全面发展。实施优质教育资源共享工程，提高义务教育均衡发展水平，推进普通高中标准化、特色化发展，加快国家职业教育试验区建设，扩大普惠幼儿园比例，鼓励连锁办园，继续推进“校安工程”建设。筹建市公共卫生服务中心，加大社区卫生服务人员培训力度，加快农村三级医疗卫生机构标准化建设。推进艺术惠民工程和精品创作工程，进一步丰富人民群众的文化生活。改造建设市区两级全民健身中心和沿河健身带、公园广场健身场地，在行政村增设户外体育设施，为城乡群众提供更多的休闲健身场所。

　　切实维护社会和谐稳定。坚持依法治市，切实加强社会管理。进一步健全群众投诉受理和权益保障机制，落实和完善重大事项社会稳定风险评估机制。加强社会治安综合治理和动态管控，深入开展“平安沈阳”创建活动。加强重点行业领域安全生产监管和专项整治，消除重点地区和重点部位的火灾隐患，有效防范重特大事故发生。加强食品药品安全监管，切实保障食品药品安全。完善市场供应保障体系，稳定市场价格，保障市场供应。做好第八届社区居民委员会换届工作，完善社区服务功能，提高社区服务水平。大力支持军队和民兵预备役部队建设，争创全国双拥模范城“七连冠”。

　　（八）切实加强政府自身建设

　　围绕进一步优化发展环境，强化依法行政，提高行政效能，努力建设人民满意的服务型政府。

　　切实提高执行力和公信力。坚持决策的科学化、民主化，使各项决策更加符合实际。各地区、各部门要进一步增强大局意识，各司其职，注重配合，不断提高政府的执行力。要强化监督考核和行政问责，确保政府各项工作得到全面落实，兑现承诺，取信于民。

　　进一步改进工作作风。各级领导干部要以身作则、率先垂范，敢于面对矛盾、善于解决问题，进一步增强加快发展的责任感、紧迫感和使命感，把心思放在抓事业上、把精力投在抓工作上、把本事用在抓发展上、把能力和水平体现在抓落实上，全心全意为企业服务、为基层服务、为百姓服务。

　　大力加强勤政廉政建设。不断提高公务员综合素质和业务能力，努力建设勤勉尽责、廉洁自律的公务员队伍。建立健全惩治和预防腐败体系的各项制度，加强对行政权力运行的监督，塑造风清气正的政务环境。

　　各位代表，沈阳的发展已经站在新的历史起点上。让我们在省委、省政府和市委的正确领导下，求真务实，锐意创新，开拓进取，埋头苦干，为实现“十二五”宏伟目标、加速沈阳老工业基地全面振兴而努力奋斗！
